# Editorial: Melatonin and biological rhythms: from bench to bedside

**DOI:** 10.3389/fnins.2023.1208878

**Published:** 2023-05-25

**Authors:** Clarissa Bueno, Fernanda Gaspar do Amaral, Karen Spruyt

**Affiliations:** ^1^Department of Neurology, Clinical Hospital of the Faculty of Medicine, University of São Paulo, São Paulo, Brazil; ^2^Department of Physiology, Federal University of São Paulo, São Paulo, Brazil; ^3^Institut National de la Santé et de la Recherche Médicale, Paris, France

**Keywords:** melatonin, circadian, rhythm, translational, metabolism, sleep

Increasingly different functions have been attributed to melatonin, the hormone produced by the pineal gland. Melatonin synthesis is regulated by the endogenous circadian timekeeping system, which makes this molecule available in circulation during the dark phase of the 24-h cycle. This profile makes this hormone an internal temporal marker, signaling the physiological night when present in the circulation. Furthermore, the presence of circulating melatonin the previous night also modulates daytime functions the following day. Besides acting as a chronobiotic and a sleep-inducing molecule, melatonin also plays roles in metabolism, cardiovascular, autonomic, and immune systems and as a potent antioxidant (Cipolla-Neto and Amaral, 2018).

The cardiometabolic role of melatonin and its relation to depressive symptoms has been investigated by Partsernyak et al. in patients with polymorbid cardiovascular pathology and metabolic syndrome. The experimental branch of this study revealed lower expression of MT1 and MT2 receptors in the epiphysis tissue of individuals with polymorbid cardiovascular pathology and metabolic syndrome, compared to controls. The clinical study addressed the impact of standard therapy of heart disease and pharmacological and psychotherapeutic treatments of depressive spectrum disorder in cardiometabolic parameters. Blood pressure circadian rhythm, and 6-sulfatoximelatonin excretion were also evaluated by the authors. The effects of circadian misalignment have already been demonstrated in several metabolic parameters as glucose tolerance, insulin and leptin levels, as well as in the cardiovascular system. Therefore, in the 24-h society we have become, it is urgent to find behavioral and other therapeutic approaches to minimize the damage.

The role of melatonin has also been revealed with its action during pregnancy and implications for fetal programming. Once melatonin diffuses easily through the placental tissue, it is an important photoperiodical signal to the fetus, capable of synchronizing the fetal physiology to mother rhythms and to the exterior environment (Reiter et al., 2014; McCarthy et al., 2019). Mendez et al. bring this subject to this Research Topic investigating the effect of melatonin supplementation on impaired endocrine, inflammatory, and gene expression variables in pregnant rats submitted to a chronodisruption protocol. Under chronic photoperiod shifting during gestation, the animals had an impact on pregnancy outcomes and disrupted circadian rhythms of locomotor activity, temperature, and heart rate. Adult female offspring had attenuated day/night differences in melatonin and corticosterone levels, pro-inflammatory cytokines, and the expression of a group of functional genes, including clock genes. Melatonin treatment during gestation had a positive impact in restoring both mother and offspring rhythms. Once again the deleterious effects of long-term chronodisruption are brought to light in aspects previously ignored, where translational studies are needed, due to the possible impact on human neurodevelopment.

Translating the results of non-human experiments to human health is a task that requires caution, especially considering that most experimental models are nocturnal animals, which change the phase relationship of melatonin and other endogenous rhythms, considering that melatonin is released at night both in diurnal and nocturnal species. Studies with primates as experimental models are limited by ethical issues, which makes the few papers published worthy of attention. We have a brief research report in this issue by Granado et al. investigating the immunoexpression of MT1 and MT2 receptors and Per1 protein in the inferior olivary nucleus at two-time points in the *Sapajus apella* monkey. The inferior olivary nucleus is involved in motor control and motor learning and the expression of melatonin receptors in motor areas has received little attention so far. Considering that motor control and coordination are impaired in several neurodegenerative diseases, elucidating the presence and profile of melatonin and clock gene expressions in these areas can open new frontiers for research.

In addition to signaling the 24-h cycle, melatonin has also the important function of being the endogenous representation of the photoperiod. Seasonal oscillations in behavioral and physiological aspects have been underestimated in human beings and the role of melatonin in human seasonality has been under debate. The influence of season on circadian phase entrainment and estimated dim-light melatonin onset has been previously reported (Zerbini et al., 2021). Melatonin levels, estimated by the degree of pineal calcification, is negatively correlated to total seasonality score, suggesting that seasonality in human behavior is related to the functioning of the pineal gland (Kunz et al., 2021). Seidler et al. deepened the discussion by studying seasonal variations in the sleep architecture of subjects attending a neuropsychiatric sleep lab. Seasonal differences between summer and winter in REM sleep and total sleep time were observed, but interestingly also an unexpected decrease in slow-wave sleep was reported in autumn. We expect that a better understanding of seasonal changes in human sleep and its relation to circulating melatonin will emerge with future research.

Decades after its discovery, there is still much to unravel about the functions of melatonin and how they are manifested in different species. The hardworking task of translating to humans not only research about the 24-h sleep/wake cycle but also other endogenous rhythms under melatonin influence has received recent impulses that we have tried to contemplate in this Research Topic. To conclude, this article collection brought cutting-edge articles, addressing newly discovered and other rediscovered roles of melatonin, which we hope will be a contribution to the field.

## Author contributions

CB contributed to the conception and writing of the original draft. KS contributed to reviewing and editing the article. All authors contributed to the finalization of the editorial. All authors contributed to the article and approved the submitted version.
